# ImageJ/Fiji ROI 1-click tools for rapid manual image annotations and measurements

**DOI:** 10.17912/micropub.biology.000215

**Published:** 2020-01-28

**Authors:** Laurent S.V. Thomas, Jochen Gehrig

**Affiliations:** 1 ACQUIFER is a division of DITABIS, Digital Biomedical Imaging Systems AG, Pforzheim, Germany; 2 Centre of Paediatrics and Adolescent Medicine, University Hospital Heidelberg, Germany

**Figure 1:**
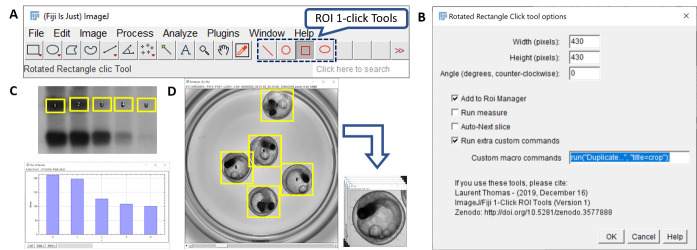
**(A)** The 1-click ROI toolset in the Fiji toolbar. Double-clicking the icon displays **(B)** the associated option window to set the ROI shapes and custom actions to execute upon clicking. **(C)** Using the rectangle 1-click tool for the quantification of bands intensity in electrophoresis gels. **(D)** Using the rectangle 1-click tool to annotate specimen position. Using a custom command (highlighted in B), the annotated regions are automatically cropped.

## Description

Manual annotation of images is considered the gold standard reference for many image-analysis tasks. Compared to automated solutions, manual annotation can be time-consuming and may be less reproducible due to human bias, especially with large datasets. However, for basic tasks like outlining a region-of-interest (ROI) and performing measurements, manual annotation is often faster and more efficient than developing a custom automated pipeline.

To facilitate manual annotations of images, we developed a set of 1-click ROI tools for ImageJ/Fiji (Schindelin *el al.* 2012; Schneider *el al.* 2012) that can be configured with predefined shapes and trigger simple tasks upon a single mouse click. Using the 1-click tools, a single mouse click on the image generates a ROI of predefined shape and size, centered on the clicked pixel. If selected in the options, the ROI is added to the ImageJ ROI Manager, the features selected in the “Set Measurements” menu are measured for this region and appended to the Results table, and if a stack is used the next image in the stack can be automatically displayed. The dimensions of the ROI as well as the optional actions to be performed can be set in the option window that is accessible by double-clicking the icon of the 1-click ROI tools (Figure 1A, B). The full toolset comprises a line, circle, rectangle and ellipse tool. In addition to setting of ROI dimensions, the line, rectangle and elliptical tool can also be rotated (see options window – Figure 1B).

The 1-click ROI tools can be used for many applications, including the quantification of intensity bands in electrophoresis gel (Figure 1C) or the annotation of cells or sub-cellular compartments. The rectangular click tool can also be used to rapidly generate bounding boxes of given dimensions, for instance to generate ground-truth annotations for object-detection algorithms (Figure 1D).

The full source code is contained in a single file, entirely written in the ImageJ macro language. Therefore, it can be easily adapted for the development of custom tools. Simple customization can be achieved by providing a user-defined macro command (typically as recorded by the macro-recorder) in the option window of the tool. The custom command will be systematically executed for new ROI generated with any of the click tools. Figure 1D illustrates this option for the automated cropping of the region of interest. Thanks to the identical size of the ROI, the resulting cropped images can directly be joined into a stack and a montage after annotation. More advanced users can also modify the source code at an indicated section, to define more complex command sequences to be executed upon click.

For further automation, keyboard shortcuts associated to custom macros can also be defined in the source code. The click tools can thus be used to generate the ROI, while the custom commands are only triggered upon keystroke, e.g. when the shape and position of the ROI is satisfactory. As an example, we defined a custom shortcut (keystroke ‘1’) to generate a stack and montage from the cropped images as in Figure 1D. The example shortcut is automatically functional once the click tools have been installed and selected. The source code contains simple indications to help defining and customizing shortcuts.

Together with custom keyboard shortcuts, the ROI 1-click tools have the potential to dramatically reduce the time spent to manually annotate images, while potentially improving the reproducibility of the annotation and processing workflow. The toolset has a broad applicability: from the annotation of cells or nuclei, organs and tissues in model organisms, to regular patterns in material science.

**Availability**

The ROI 1-click tools can be installed in Fiji by activating the *ROI 1-click tools* update site.

In ImageJ, the tools can be installed by copying the macro file in the subfolder *“macros/toolsets”* of ImageJ.

Source code and documentation are available on GitHub (https://github.com/LauLauThom/Fiji-RoiClickTools).

A video tutorial is available on YouTube (https://youtu.be/ZPS78T_-gUs).

The gel image for figure 1C is an ImageJ sample image (credit NIH). The image for figure 1D is available on Zenodo (Gierten and Gehrig 2019).
